# Overexpression of 14-3-3ζ primes disease recurrence, metastasis and resistance to chemotherapy by inducing epithelial-mesenchymal transition in NSCLC

**DOI:** 10.18632/aging.204188

**Published:** 2022-07-22

**Authors:** Lingyun Wei, Nan Hu, Mingxiang Ye, Zhilong Xi, Zhen Wang, Lei Xiong, Nan Yang, Yi Shen

**Affiliations:** 1 Department of Cardiothoracic Surgery, Jinling Hospital, Nanjing University School of Medicine, Nanjing 210002, China; 2 Department of Stomatology, The First Medical Center of PLA General Hospital, Beijing 100853, China; 3 Department of Respiratory Medicine, Jinling Hospital, Nanjing University School of Medicine, Nanjing 210002, China; 4 Department of Radiation Oncology, Jinling Hospital, Nanjing University School of Medicine, Nanjing 210002, China

**Keywords:** non-small cell lung cancer, 14-3-3ζ, epithelial-mesenchymal transition, tumor recurrence, apoptosis resistance

## Abstract

The prognosis of non-small cell lung cancer (NSCLC) is disappointing because disease recurrence and distant metastasis inevitably occurred. The aim of the present study is to identify novel biomarkers predicting tumor recurrence and metastasis. The 14-3-3ζ protein has been extensively described as a tumor promoter in a panel of solid tumors, including NSCLC. However, there is a big gap regarding the knowledge between 14-3-3ζ and NSCLC recurrence. In this study, we found that overexpression of 14-3-3ζ was more frequent in NSCLC tumor tissues with tumor recurrence. By using scratch healing assay and transwell assay, we demonstrated that NSCLC cells with high expression of 14-3-3ζ gained increased motile and invasive capacity, whereas siRNA-mediated knockdown of endogenous 14-3-3ζ abrogated cancer cell dissemination. Intriguingly, we found that NSCLC cells underwent epithelial-mesenchymal transition (EMT) after the induction of 14-3-3ζ *in vitro* and *in vivo*. These findings could be readily recaptured in clinical setting since we showed that NSCLC tumor specimen with high expression of 14-3-3ζ revealed biological features of EMT. Overexpression of 14-3-3ζ also enhanced the phosphorylation of Akt and promoted the proliferation of NSCLC cell lines. In agreement with this notion, we reported that NSCLC cells with high expression of 14-3-3ζ became resistant to chemotherapy-induced apoptosis. These findings strongly suggested that 14-3-3ζ as a novel biomarker predicting risks of disease recurrence and screening 14-3-3ζ status may be a promising approach to select patients who experienced high risks of cancer recurrence and resistance to chemotherapy.

## INTRODUCTION

Lung cancer is one of the leading causes of cancer-associated death worldwide. In China, more than 733,000 new cases were diagnosed each year, causing more than 610,000 cases of death [1]. Non-small cell lung cancer (NSCLC) is the most common histological type of lung cancer, in which it accounts for approximately 85% of lung cancer cases. Since the discovery of oncogenic driver genes and immune checkpoints, numerous novel therapeutic approaches for NSCLC at advanced stage have been well established. Epidermal growth factor receptor (EGFR) and anaplastic lymphoma kinase (ALK) are two paradigms of cancer driven genes that provide highly efficient therapeutic targets [2–5]. Tyrosine kinase inhibitors significantly improve the progression free survival (PFS) and quality of life for NSCLC patients with distinct genetic alterations. In contrast, the anti-PD-1 (programmed cell death-1)/PD-L1 (programmed cell death ligand-1) immunotherapy has become standard therapy for NSCLC patients without oncogenic driver mutations [6–8]. Patients who received anti-PD-1/PD-1 immunotherapy have achieved robust clinical benefits and their overall survival (OS) has been largely extended [9–12]. However, these novel treatment options are not recommended for NSCLC patients at early stage. Surgical resection remains the backbone for early stage (stage I-IIIA) NSCLC. Unfortunately, the long-term survival of early stage NSCLC patients, even after surgery, is rather disappointing. For instance, the 5-year OS ratio for patients with stage I NSCLC is only 60–70%, whereas it falls to 35–40% for those with stage II tumors [13]. For stage III NSCLC patients who received platinum-based palliative chemotherapy, an average PFS of only 8–10 months was reached [14]. Post-operational chemotherapy and radiation therapy tended to prohibit disease recurrence and metastasis. However, only a small proportion of patients benefit from these treatments and most patients develop resistance to chemotherapeutic agents, thus, there is urgent clinical needs to establish biomarkers to identify NSCLC patients at high risk of disease recurrence and resistance to anti-tumor treatment.

The 14-3-3 proteins are highly conserved in mammals and play crucial roles in a wide range of biological events [15]. Briefly, 14-3-3 proteins bind to phosphorylated serines or threonines primarily located in the conserved motifs RSXpSXP or RXXXpSXP of over 200 protein targets. The binding leads to altered subcellular localization, phosphorylation status, and enzymatic activity of corresponding client proteins, which are implied in signaling transduction, cell proliferation, cell adhesion, cell apoptosis and tumorigenesis [16–18]. There are seven isoforms of 14-3-3 proteins in human genome, in which 14-3-3σ is considered as a putative tumor suppressor [19]. In contrast, 14-3-3β, γ, ε, ζ, η, and τ isoforms are implicated in tumorigenesis [20–22]. The 14-3-3 proteins have distinct tissue distribution patterns and isoform-specific roles. For example, 14-3-3γ is predominantly expressed in the brain, muscle and heart, but it is absent in peripheral blood leukocytes [23]. Loss of 14-3-3σ due to hypermethylation is reported in small cell lung cancer, but its expression is not affected in NSCLC. Previous studies have shown that 14-3-3ζ binds to Cdc25 and induces G2/M arrest, whereas 14-3-3β promotes cell proliferation and mitosis [24, 25]. These lines of evidence strongly suggest that elaboration of 14-3-3 protein contributes to cancer cell growth. The ζ isoform of 14-3-3 protein has attracted increasing interest these years because its elevated expression is detected in a variety of cancers [18]. 14-3-3ζ, also known as YWHAZ, has been reported to interact with multiple cell survival signaling and exerted anti-apoptotic function. Mechanistically, 14-3-3ζ binds to the p85 regulatory subunit of PI3K, leading to the phosphorylation of Akt, a key executor of cell proliferation, and inactivation of tumor suppressors like p53 and p21 [26]. Overexpression of 14-3-3ζ also enables resistance to anoikis and promotes non-invasive breast cancer progressed into invasive breast cancer [27]. Although there are several lines of evidence supporting the involvement of 14-3-3ζ during NSCLC tumorigenesis and prognosis, however, evidence connecting 14-3-3ζ directly to NSCLC relapse and metastasis is lacking and warrants further experimental research [28].

The epithelial–mesenchymal transition (EMT) is a biological process that allows polarized epithelial cells to undergo multiple biochemical changes, in which cells assume a mesenchymal phenotype to gain enhanced migratory capacity and subsequently acquisition of invasiveness to adjacent tissue [29]. Activation of the EMT program has been proposed as the critical mechanism for the acquisition of metastatic phenotypes. Although the full spectrum of signaling that contribute to EMT has not been well established, a number of distinct molecules that initiate and complete EMT, including activation of transcriptional factors, expression of specific cell-surface proteins and reconstruction of cytoskeletal proteins, have been identified [30]. Intriguingly, aberration of oncogenes also provokes EMT signaling and facilitates subsequent aggressive dissemination. Genetic profiling of cancer cells that are resistance to anti-cancer reagents also indicates signatures of EMT [31]. To this end, there are compelling clinical needs to identify potent EMT regulators aiming to prevent cancer metastasis and overcoming resistance to chemotherapy.

The goal of this study was to discover the significance of 14-3-3ζ during cancer recurrence and metastasis in surgical resectable early stage NSCLC patients. Herein, we demonstrated that 14-3-3ζ and NSCLC recurrence converge at EMT. We provided preliminary evidence showing 14-3-3ζ overexpression confers poor prognosis and is a novel marker predicting disease recurrence. Overexpression of 14-3-3ζ promotes cells undergoing EMT, as a consequence, leading to cancer cell metastasis. We also demonstrated that overexpression of 14-3-3ζ increased the phosphorylation of Akt and resulted in resistance to chemotherapeutic agents. Taken together, the present study highlighted 14-3-3ζ as a prognostic biomarker predicting surgery outcomes in NSCLC patients at early stage. Screening the expression of 14-3-3ζ could be a promising approach to select patients who experienced high risks of cancer recurrence and resistance to chemotherapy.

## RESULTS

### Bioinformatic analysis of 14-3-3ζ in NSCLC

**Figure 1:**
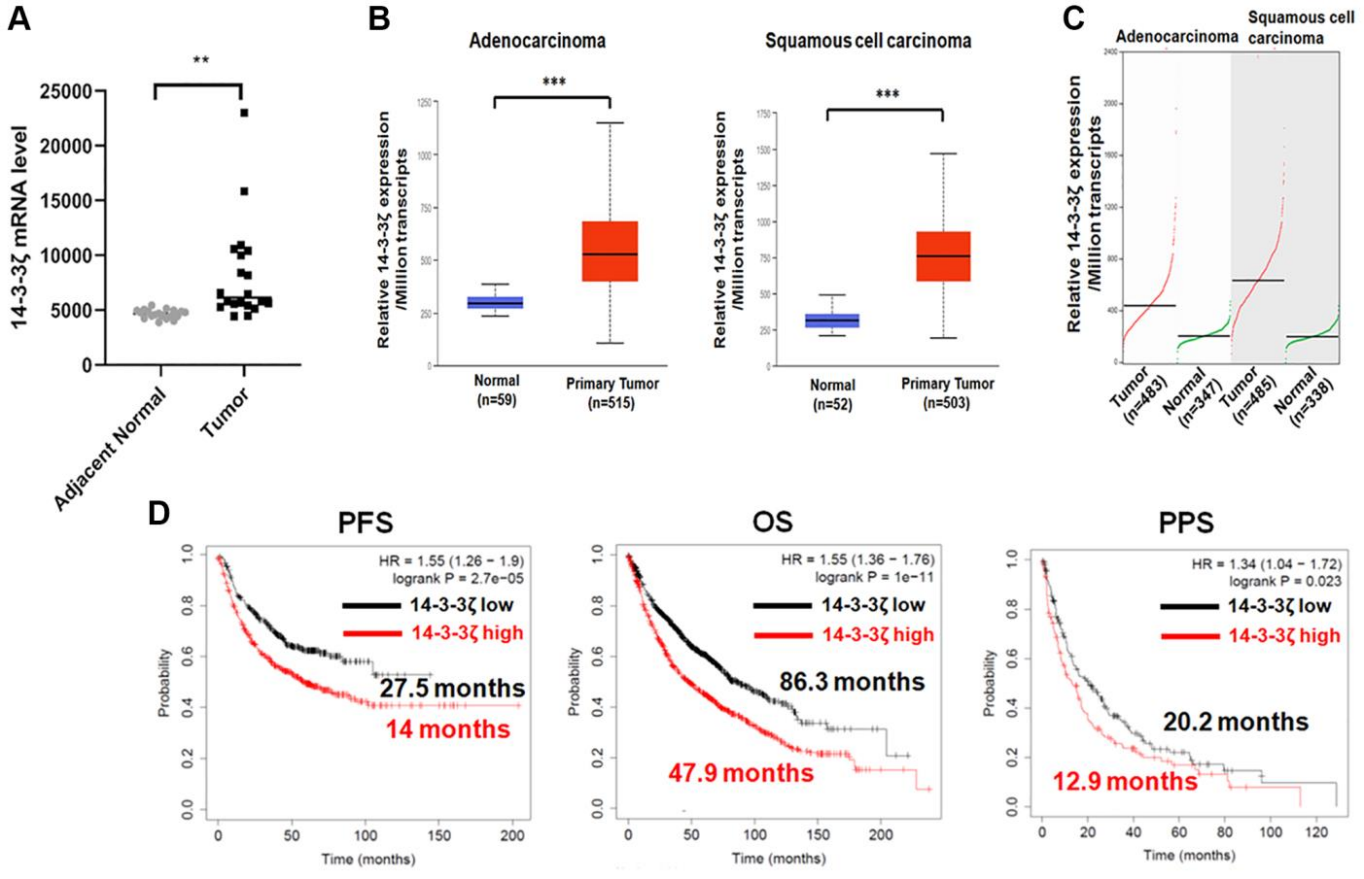
**Bioinformatic analysis of 14-3-3ζ mRNA expression in NSCLC.** (**A**) Expression of 14-3-3ζ mRNA in tumor tissues and adjacent non-tumor tissues (^**^P < 0.01 vs. adjacent normal lung tissues). (**B** and **C**) Expression of 14-3-3ζ transcripts in lung adenocarcinoma or squamous cell carcinoma and in corresponding normal lung tissue from UALCAN (**B**) and GEPIA (**C**) database. Relative expression of 14-3-3ζ was presented as the amount of 14-3-3ζ mRNA among one million transcripts (^***^P < 0.01 vs. normal lung tissue). (**D**) Log-rank survival analysis of NSCLC patients with different status of 14-3-3ζ. The red line indicates NSCLC patients with high expression of 14-3-3ζ, and the black line indicates those with low expression. Patients’ progression free survival (PFS), overall survival (OS), and post-progression survival (PPS) data in each cohort were presented along with the survival curve.

In order to verify the correlation between 14-3-3ζ expression and prognosis in NSCLC, we used TCGA, GEPIA and GEPIA online bioinformatic tools. As shown in Figure 1A, 14-3-3ζ mRNA level was markedly increased in NSCLC tumor samples, in comparison with adjacent non-tumor tissues. Further in-depth sub-group analysis confirmed that 14-3-3ζ expression universally elevated in NSCLC, regardless of histological types (Figure 1B and 1C). In agreement with these observations, the log-rank survival analysis indicated that high level of 14-3-3ζ in patients with NSCLC conferred unfavorable PFS and OS with the hazard ratio (HR) of 1.34 (95% CI: 1.26–1.9) and 1.5 (95% CI: 1.36–1.76), respectively (Figure 1D). Of noted, the median PFS (14.3 months vs. 27.5 months) and median OS (47.9 months vs. 86.3 months) in the 14-3-3ζ high cohort was markedly lower than that in the 14-3-3ζ low cohort. After disease progression, patients with high expression of 14-3-3ζ tended to have a shorter post-progression survival (PPS, 12.9 months vs. 20.2 months) with the HR value of 1.34 (95% CI: 1.04–1.72). Thus, high expression of 14-3-3ζ is an unfavorable prognostic biomarker for NSCLC.

### Overexpression of 14-3-3ζ predicts disease recurrence in early stage NSCLC

**Figure 2:**
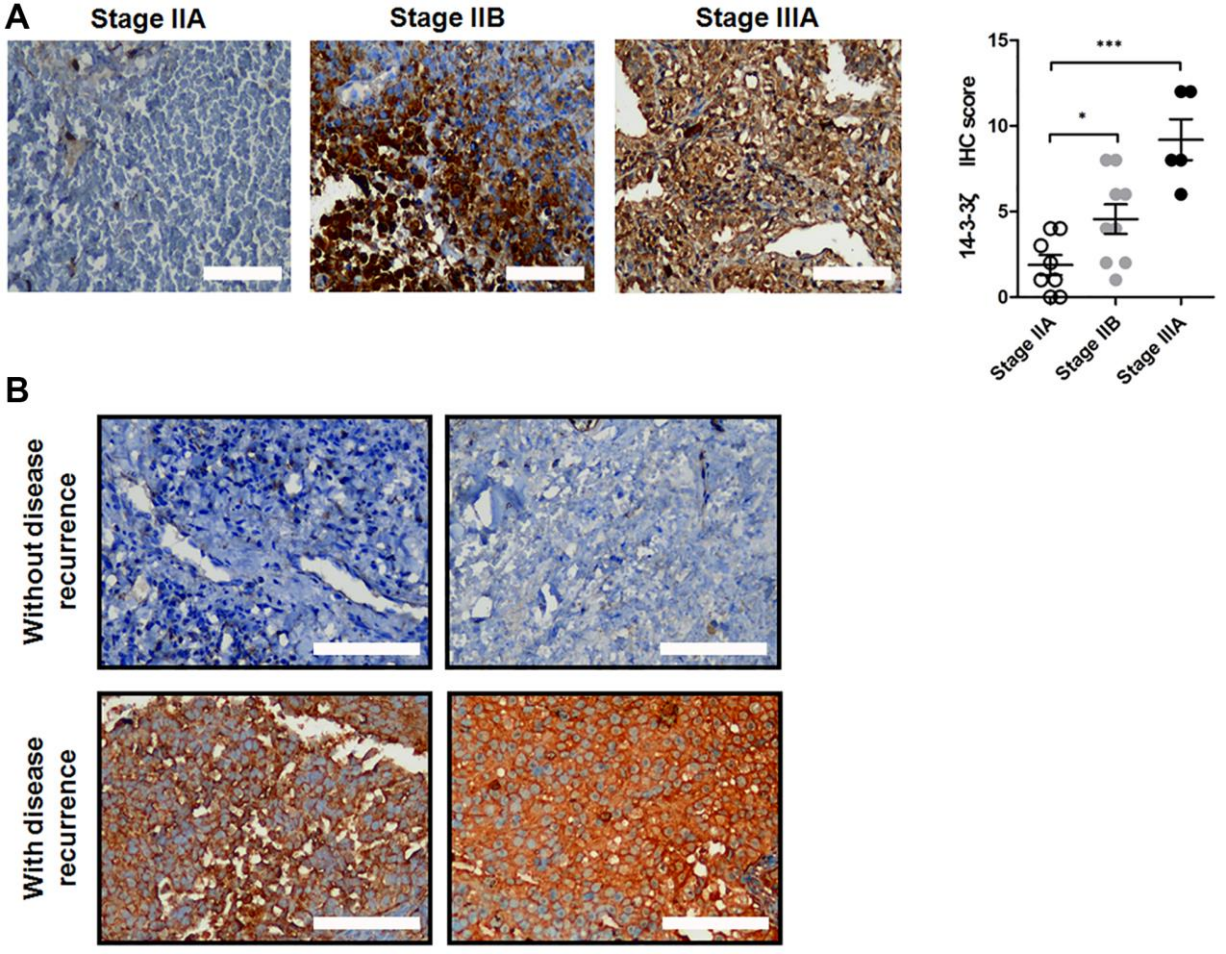
**Expression of 14-3-3ζ protein in surgical resected NSCLC samples.** (**A**) Representative IHC staining of 14-3-3ζ protein in NSCLC tissues at different clinical stage. The IHC score of 14-3-3ζ in each group was analyzed (^*^P < 0.05 vs. stage IIA, ^***^P < 0.01 vs. stage IIA). Scale bar = 200 μm. (**B**) Representative IHC images of 14-3-3ζ protein in patients without (upper panel) and with (lower panel) disease recurrence. Scale bar = 200 μm.

Having demonstrated that 14-3-3ζ negatively correlates with the prognosis in NSCLC patients, we next determined whether overexpression of 14-3-3ζ confers disease recurrence in early stage NSCLC patients who received tumor surgical resection. We collected paired surgical resected NSCLC specimens and adjacent non-tumor tissues from 2019 to 2020. These patients were well balanced and their baseline characteristics were listed in Table 1. The expression of 14-3-3ζ was strongly positive in approximately 70% (*n* = 16/22) of patients’ NSCLC specimens, which is consistent with previous reports of 14-3-3ζ overexpression in 80% of NSCLC cases. Strikingly, the expression of 14-3-3ζ was more profound in NSCLC at stage IIB and stage IIIA, and their IHC scores of 14-3-3ζ were significantly higher than that in the stage IIA group (Figure 2A, ^*^P < 0.05 vs. stage IIA, ^***^P < 0.001 vs. stage IIA). Among the 14-3-3ζ overexpressing patients, four of the 16 cases had disease recurrence with metastasis, whereas none of the 6 cases whose tumors did not overexpress 14-3-3ζ developed disease recurrence (Figure 2B). Of noted, positive staining of 14-3-3ζ was mostly observed in the cytoplasm of NSCLC cancer cells. Thus, overexpression of 14-3-3ζ in this small cohort positively correlated with disease recurrence and metastasis, suggesting that 14-3-3ζ may be a biomarker predicting disease progression and metastasis in early stage NSCLC.

**Table 1:** Baseline characteristics of patients with early stage NSCLC.

**Characteristics**	***n* (%)**
**Age**	
Average	62.4 (56.8–70.5)
≥65 years	10 (45.5%)
<65 years	12 (54.5%)
**Gender**	
Male	8 (36.4%)
Female	14 (63.6%)
**Smoking status**	
Smokers	6 (27.3%)
Non-smokers	16 (82.7%)
**ECOG score**	
0	18 (81.8%)
1	2 (9.1%)
2	2 (9.1%)
**Clinical stage**	
IIA	8 (36.4%)
IIB	9 (41.0%)
IIIA	5 (22.6%)
**Histology**	
Adenocarcinoma	16 (72.7%)
Squamous	5 (22.6%)
Adeno-squamous	1 (4.7%)
**14-3-3ζ expression**	
Positive	16 (72.7%)
Negative	6 (27.3%)
**Disease recurrence**	
Yes	4 (18.2%)
No	18 (81.8%)

### 14-3-3ζ increases cancer cell mobility

**Figure 3:**
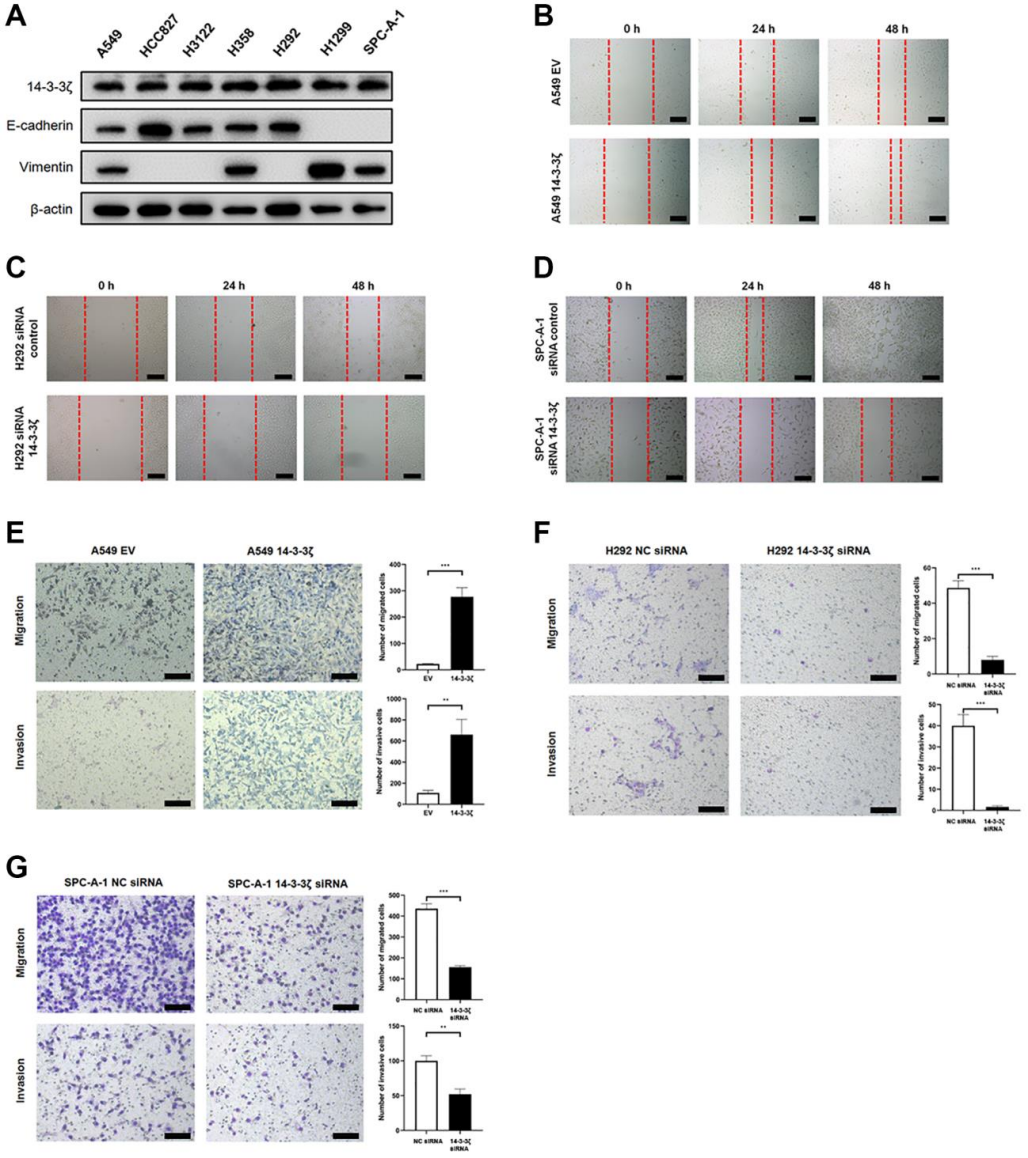
**The 14-3-3ζ promotes NSCLC cell mobility, migration and invasion.** (**A**) Western blot analysis of endogenous expression of 14-3-3ζ, E-cadherin and vimentin in a panel NSCLC cell lines. β-actin was used as equal loading control. (**B**–**D**) Evaluation of 14-3-3ζ on NSCLC cell mobility by wound healing assay. After indicated treatment, cells were seeded into 6-well plates and an artificial wound was created. Photographs were taken at indicated time points and representative images were shown. Scale bar = 100 μm. (**E**, **F**) The 14-3-3ζ was either overexpressed (**E**) or suppressed (**F**, **G**) in NSCLC cell lines, and the resultant cells were tested for migration and invasion capacity by transwell assay, respectively. Representative images at different time points were shown. Scale bar = 100 μm. The number of migrated and invaded cells was calculated and analyzed for statistical significance. ^**^P < 0.01. ^***^P < 0.001.

Given that high expression of 14-3-3ζ stratified NSCLC patients at high risk of disease recurrence, we anticipated that overexpression of 14-3-3ζ may enhance cell mobility, as a consequence, leading to cancer cell metastasis. To test this hypothesis, we detected the endogenous expression of 14-3-3ζ protein in a panel of NSCLC cell lines. All the tested cell lines (A549, HCC827, H3122, H358, H292, H1299 and SPC-A-1) expressed different level of 14-3-3ζ protein, in particularly H292 and SPC-A-1 cells expressed relatively higher amount of 14-3-3ζ protein level, whereas the endogenous level of 14-3-3ζ protein in A549 cells was relatively low (Figure 3A). Thus, we selected these three cell lines and used molecular approaches to manipulate 14-3-3ζ expression and investigate its potential metastasis promoting significance in our following experiments.

By utilizing scratch healing assay, we found that ectopic overexpression of 14-3-3ζ facilitated the scratch healing capacity in A549 cells (Figure 3B). In contrast to cells transfected with siRNA control, siRNA-mediated knockdown of 14-3-3ζ readily ameliorated such healing in H292 cells (Figure 3C). Similar findings were replicated in SPC-A-1 cells, when endogenous 14-3-3ζ expression was suppressed (Figure 3D). These results strongly indicated that 14-3-3ζ enables cancer cells to gain increased motile capacity, which in return contributes to cancer cell metastasis.

To collaborate with these findings, we performed transwell migration and invasion assays. As illustrated in Figure 3E, the A549 cells infected with empty vector (EV, A549 EV) lentivirus supernatant minimally migrated the chamber, whereas the migration activity was dramatically increased when the A549 cells were infected with the 14-3-3ζ (A549 14-3-3ζ) lentivirus supernatant. In consistent with this notion, the A549 cells overexpressing 14-3-3ζ exhibited sharply increased invasive capacity. However, inhibition of 14-3-3ζ in H292 (Figure 3F) and SPC-A-1 (Figure 3G) cells led to significantly decreased migration and invasion activity. Thus, 14-3-3ζ is a key regulator of cell mobility in NSCLC. Overexpression of 14-3-3ζ is a frequent event in NSCLC, indicating that screening 14-3-3ζ expression could be used as a biomarker to predict cancer metastasis and disease recurrence.

### 14-3-3ζ induces cancer cell undergoing EMT

**Figure 4:**
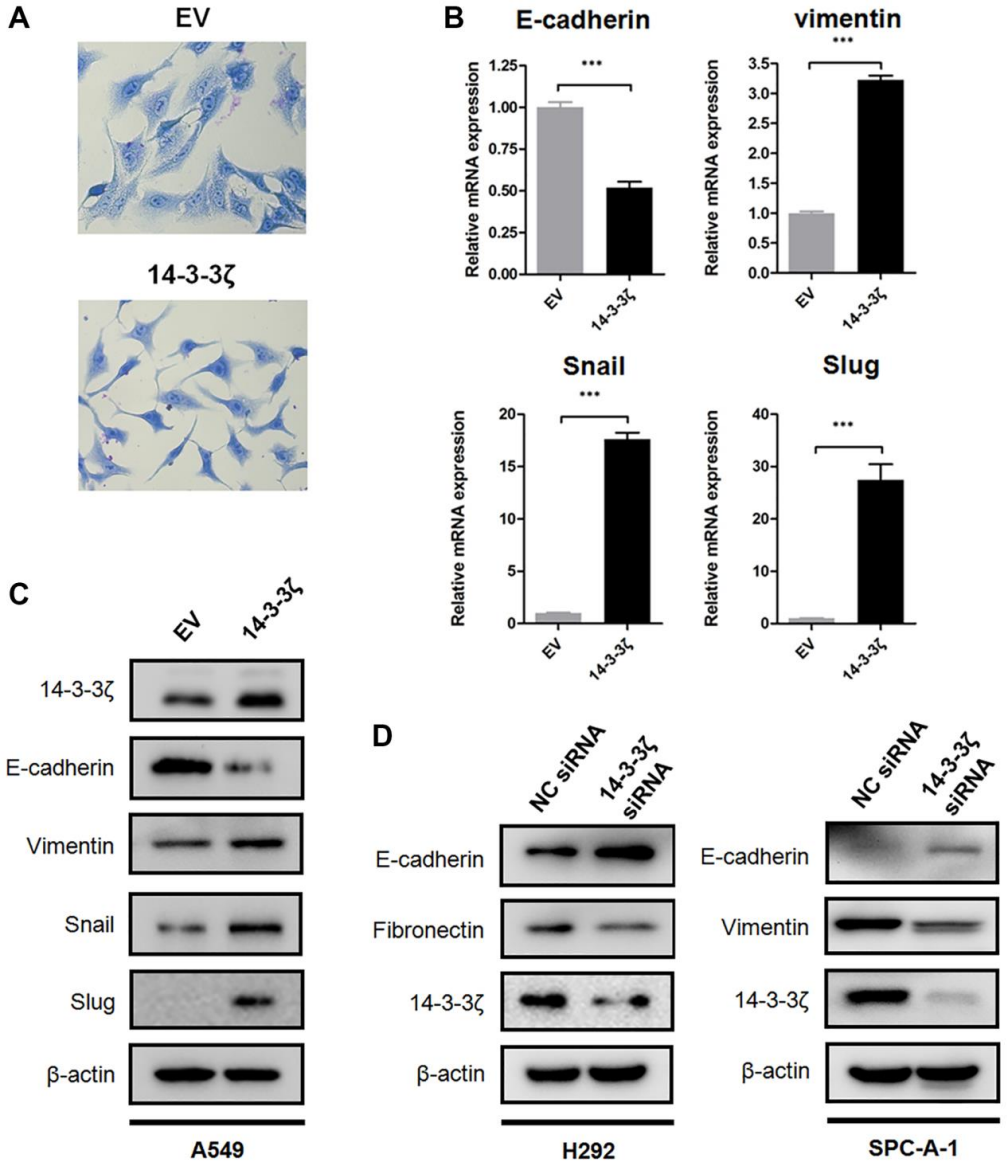
**14-3-3ζ promotes NSCLC cells undergoing EMT.** (**A**) Representative images of morphological changes in A549 cells overexpressing 14-3-3ζ. Scale bar = 200 μm. (**B**) Real time qPCR analysis of EMT biomarkers and transcriptional factors following upregulating 14-3-3ζ in A549 cells (^***^P < 0.001 vs. EV). (**C**) Immunoblotting of E-cadherin, vimentin, Snail and Slug protein in the A549 EV and A549 14-3-3ζ cells, respectively. β-actin was used as equal loading control. (**D**) Immunoblotting of EMT biomarkers in NSCLC cells transfected with indicated siRNA. β-actin was used as equal loading control.

Next, we explored potential mechanism that underlies increased cell mobility. We noticed that A549 cells (relatively low endogenous 14-3-3ζ) underwent robust morphological alterations after the induction of 14-3-3ζ. Briefly, the A549 EV cells exhibited canonical epithelial features, including paving-stone appearance, tight cell-cell adhesion, and cell polarity. In contrast, the A549 14-3-3ζ cells tended to scatter and switched into spindle-like appearance (Figure 4A). These morphological alterations reflected cells undergoing a phenotype conversion, known as EMT, which has been extensively documented during cancer metastasis.

To validate whether EMT is a consequence of 14-3-3ζ overexpression, we examined the expression of biomarkers and transcriptional factors involved in EMT. There is emerging evidence showing that E-cadherin is predominantly expressed on the cell membrane of epithelial cells and it is a crucial protein for maintaining cell adhesion junction. In contrast, overexpression of vimentin is associated with the acquisition of mesenchymal features and metastatic capacity [29]. Loss of E-cadherin and increased expression of vimentin represent the hallmarks of EMT and is frequently detected in various cancers. To this end, we assessed the expression of E-cadherin and vimentin in A549 EV and A549 14-3-3ζ cells, respectively. Notably, qPCR assay indicated that overexpression of 14-3-3ζ suppressed mRNA transcription of E-cadherin, whereas it increased the mRNA expression of vimentin. This EMT-promoting effect was accompanied by enhanced transcription of two E-cadherin suppressors, Snail and Slug (Figure 4B). In agreement with these findings, Western blot analysis suggested that overexpression of 14-3-3ζ in A549 cells readily suppressed the expression of E-cadherin at protein level, while it induced a sharp increase in mesenchymal biomarker vimentin and EMT-associated transcriptional repressors (Figure 4C). Knockdown of 14-3-3ζ in H292 and SPC-A-1 cells elicited an opposite effect in comparison with that in 14-3-3ζ overexpressing cells. As shown in Figure 4D, siRNA-mediated suppression of 14-3-3ζ in H292 and SPC-A-1 cells partially rescued E-cadherin expression. The expression of mesenchymal biomarkers, including Fibronectin and vimentin, was reduced following the inhibition of 14-3-3ζ.

**Figure 5:**
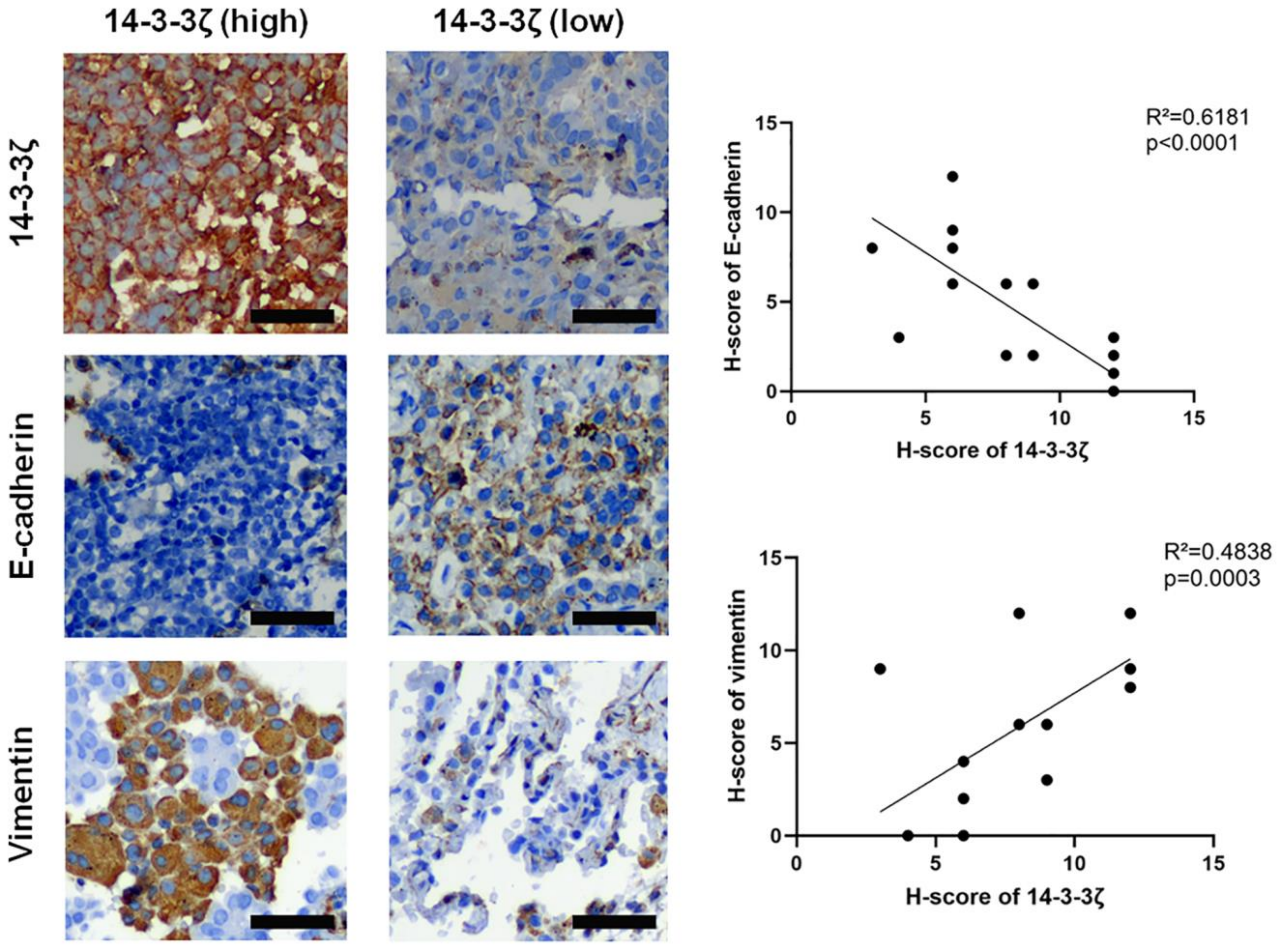
**Representative IHC images of E-cadherin and vimentin in NSCLC tissues with different status of 14-3-3ζ.** Scale bar = 200 μm. The linear regression analysis was used to determine the correlation between 14-3-3ζ, E-cadherin and vimentin, respectively.

Furthermore, the correlation between 14-3-3ζ and EMT was confirmed in NSCLC specimens. Figure 5 showed representative IHC images of E-cadherin and vimentin staining in NSCLC with different status of 14-3-3ζ. It was obvious that NSCLC overexpressing 14-3-3ζ expressed relatively lower level of E-cadherin, but higher level of vimentin. In contrast, NSCLC negative or weakly positive for 14-3-3ζ repressed the expression of vimentin while restored the expression of E-cadherin. The linear regression analysis of IHC score in NSCLC cases also confirmed a negative correlation between 14-3-3ζ and E-cadherin, however, a positive correlation between 14-3-3ζ and vimentin was noticed. These results indicated the 14-3-3ζ–EMT interaction also existed in clinical setting, and 14-3-3ζ is the key component that drives EMT and promotes cancer cell metastasis in NSCLC.

### Overexpression of 14-3-3ζ promotes metastasis *in vivo*

**Figure 6:**
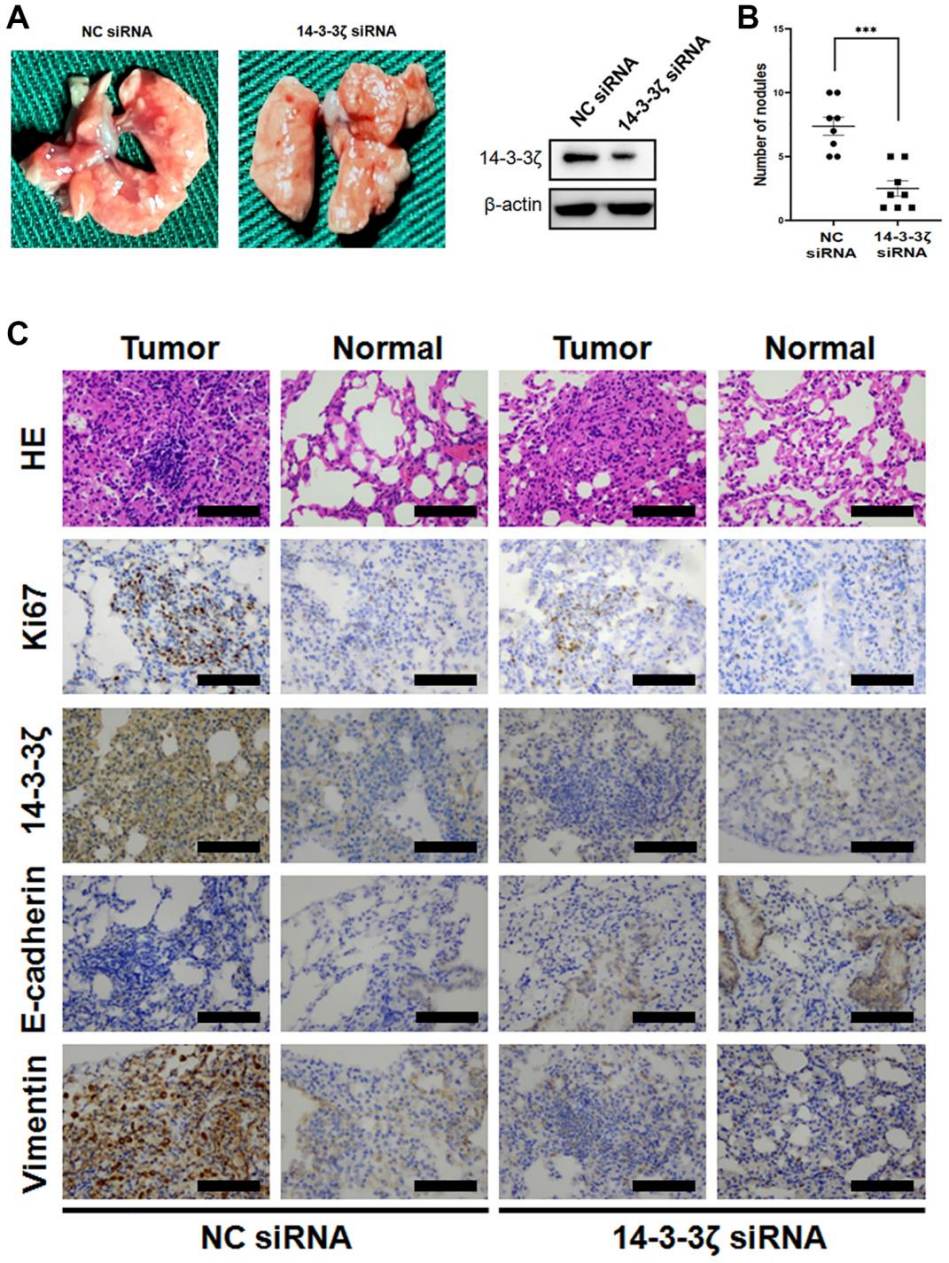
**Overexpression of 14-3-3ζ accelerates LLC cell lung metastasis *in vivo*.** (**A** and **B**) The LLC cells were transfected with siRNA targeting 14-3-3ζ or negative control. After indicated treatment, cells were injected into C57BL/6J mice through the medial tail vein. At the end of experiment, mouse lungs were carefully isolated and metastatic lung nodules were calculated. (**C**) Representative histological and IHC images of mouse lungs after LLC cells tail vein injection. The metastatic LLC nodules and adjacent normal lungs were evaluated for Ki67, 14-3-3ζ, E-cadherin and vimentin expression. Scale bar = 200 μm.

To validate the metastasis-promoting and EMT-inducing capacity of 14-3-3ζ *in vivo*, we evaluated the effect of 14-3-3ζ on Lewis lung cancer (LLC) cell mobility in C57BL/6J mice by injecting the tail vein with indicated cells. At necropsy, numerous superficial white opacities appeared in mice injected with NC siRNA LLC cells, however, the number of metastatic patches was markedly decrease when the expression of 14-3-3ζ was suppressed (Figure 6A and 6B).

HE staining of the mice lung showed multiple tumor cell foci invading the bronchioles and alveolar ducts. These LLC cells were strongly positive for 14-3-3ζ and the proliferative biomarker Ki67. In agreement with this notion, the expression of E-cadherin in LLC NC siRNA cells was very weak, while the cells expressed high level of vimentin (Figure 6C). The lung sections of the 14-3-3ζ siRNA mice group tended to elicit an opposite presentation, in which the number and the boundary edge of the tumor cell foci were reduced. IHC staining suggested that knockdown of 14-3-3ζ resulted in decreased LLC cell proliferation and the restoration of E-cadherin expression (Figure 6C). Thus, targeting 14-3-3ζ is sufficient to prevent cancer cell metastasis *in vivo*, probably through suppressing EMT.

### 14-3-3ζ promotes cell proliferation and contributes to resistance to chemotherapy

**Figure 7:**
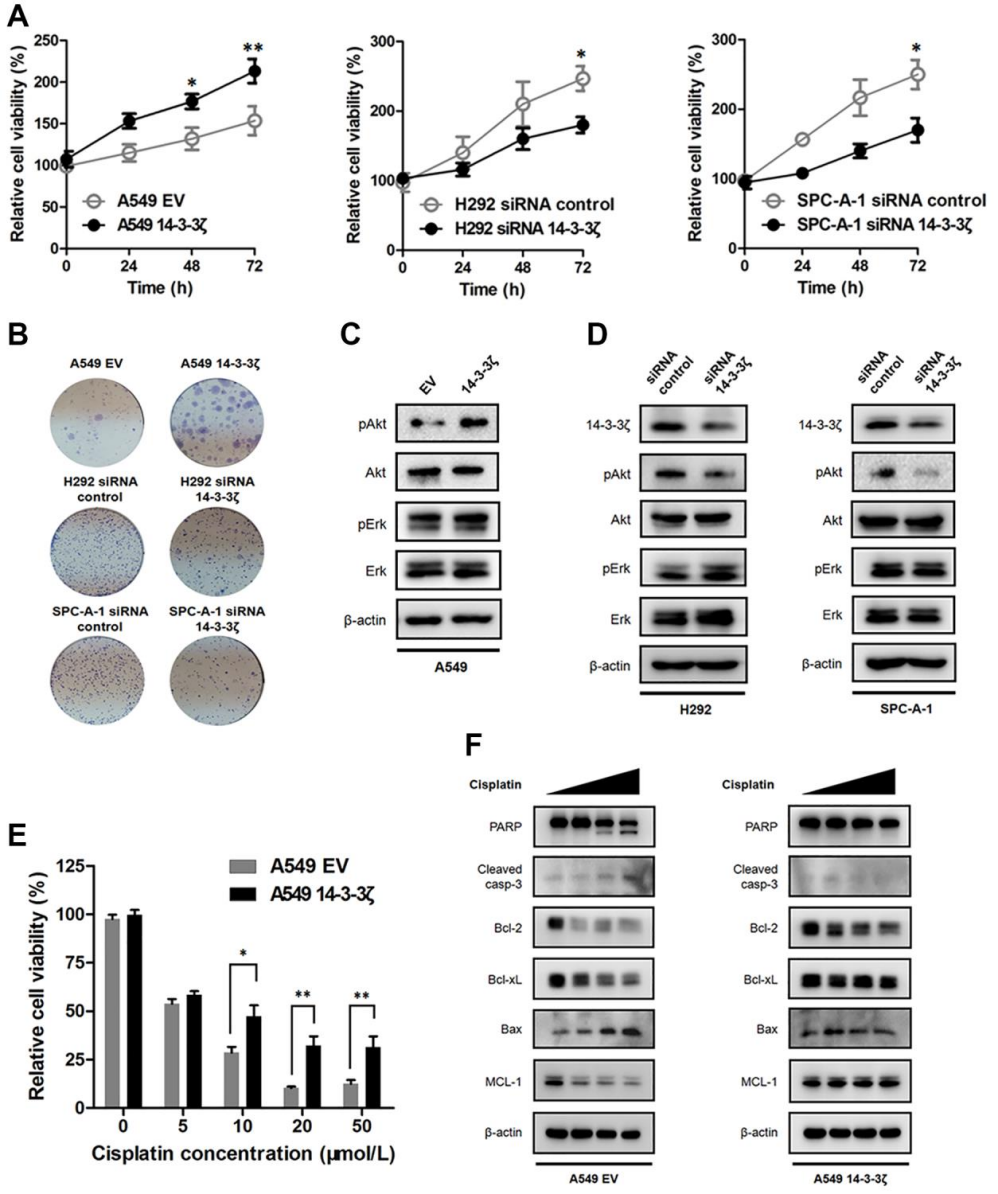
**Effect of 14-3-3ζ on NSCLC proliferation and sensitivity to chemotherapy.** (**A**) NSCLC cells with different status of 14-3-3ζ were seeded in 96-well plate to evaluate their proliferative activity at indicated time points by the MTT assay. ^*^P < 0.05, ^**^P < 0.01. (**B**) Representative images of cell colony assays after regulating the expression of 14-3-3ζ in A549, H292 and SPC-A-1 cells, respectively. (**C** and **D**) Western blot analysis of the phosphorylation status of Akt and Erk in NSCLC cells with different status of 14-3-3ζ. β-actin was used as equal loading control. (**E**) Cells were treated with increasing concentrations of cis-platin for 24 h. After indicated treatment, cell viability was measured by MTT assay. ^*^P < 0.05 vs. A549 EV group and ^**^P < 0.01 vs. A549 EV group, respectively. (**F**) Western blot analysis of apoptosis-related proteins after cis-platin treatment in A549 EV and A549 14-3-3ζ cells. β-actin was used as equal loading control.

Apart from increased cell mobility, we found that the expression magnitude of 14-3-3ζ confers the proliferative activity of cancer cells. The MTT assay showed that the A549 14-3-3ζ cells displayed increased cell viability in comparison with the A549 EV cells. In contrast, knockdown of endogenous 14-3-3ζ in H292 and SPC-A-1 cells significantly suppressed their proliferation (Figure 7A). This proliferation promoting effect was durable and could be readily recaptured in colony formation assay, in which the A549 cells overexpressing 14-3-3ζ formed abundant cell colonies. However, transfection with 14-3-3ζ siRNA resulted in the suppression of colony formation in H292 and SPC-A-1 cells, respectively (Figure 7B).

We next determined how 14-3-3ζ promotes cell proliferation. There is large body of evidence showing 14-3-3ζ interacts with multiple signaling pathways involved in cell proliferation, therefore, we evaluated the phosphorylation status of Akt and Erk after the disruption of 14-3-3ζ. Of noted, 14-3-3ζ elicited different impacts on the phosphorylation status of Akt and Erk, two key molecular executors implicated in the PI3K/Akt and MAPK/Erk signaling. We found that the phosphorylation of Akt was enhanced when cells overexpressed 14-3-3ζ, whereas the phosphorylation of Erk was not changed regardless of 14-3-3ζ status (Figure 7C). Knockdown of endogenous 14-3-3ζ attenuated the phosphorylation of Akt, but not Erk, in both H292 and SPC-A-1 cells (Figure 7D). To this end, it is reasonable to conclude that 14-3-3ζ selectively interacted with the PI3K/Akt signaling, as a consequence, leading to increased Akt phosphorylation and promoting cancer cell proliferation. Finally, we determined the biological significance of 14-3-3ζ in NSCLC treatment. We assessed the sensitivity to cis-platin in A549 EV and A549 14-3-3ζ cells, respectively. The MTT assay indicated cis-platin treatment dose-dependently suppressed the survival of A549 EV cells, whereas the A549 14-3-3ζ cells were less sensitive to such killing (Figure 7E). In agreement with this notion, Western blot analysis showed that 14-3-3ζ readily abrogated the intrinsic apoptosis pathway. As shown in Figure 7F, cis-platin treatment induced A549 EV cells undergoing apoptosis, which was supported by the finding that the anti-apoptotic protein Bcl-2, Bcl-xL and MCL-1 dramatically declined following the treatment. Moreover, the pro-apoptotic protein Bax and the apoptosis executor caspase-3 and PARP markedly increased after cis-platin treatment. Intriguingly, the A549 cells became resistant to cis-platin treatment when ectopic 14-3-3ζ encoding plasmid was introduced. The anti-apoptotic protein Bcl-2, Bcl-xL and MCL-1 became stabilized and the pro-apoptotic protein Bax failed to respond to cis-platin in the A549 14-3-3ζ cells. Furthermore, cis-platin-induced activation of caspase-3 and PARP was also prohibited after the induction of 14-3-3ζ (Figure 7F). Collectively, these results suggested that overexpression of 14-3-3ζ disrupted the intrinsic apoptosis pathway and promoted resistance to chemotherapy in NSCLC.

## DISCUSSION

In the present study, we identified the overexpression of 14-3-3ζ as a crucial event implicating poor prognosis and disease recurrence in NSCLC at early stage. Overexpression of 14-3-3ζ promotes tumor progression and metastasis by inducing EMT and confers survival advantage under stress conditions, whereas knockdown of 14-3-3ζ suppresses cancer cell migration and invasion. These findings prompt clinicians to select NSCLC patients who may have risks of disease recurrence and resistance to chemotherapy, as a consequence, more aggressive treatment strategies are recommended in NSCLC patients with high expression of 14-3-3ζ.

The 14-3-3 proteins have been extensively studied for their potential tumor promoting or tumor suppressing functions. The 14-3-3ζ isoform has been recognized as a negative prognostic factor for breast cancer, in which it associates with decreased disease-free survival and high risks of cancer recurrence [32]. By using online bioinformatic tools, we found that the expression of 14-3-3ζ universally increased in NSCLC, regardless of histological types. We also reported 14-3-3ζ as a biomarker for predicting prognosis and disease recurrence in early stage NSCLC patients who received surgical resection. These findings are consistent with previous reports and may have a broad spectrum of clinical significance [33, 34]. Identifying NSCLC patients with overexpression of 14-3-3ζ may indicate high risk of disease recurrence, and these patients would require subsequent intensive or adjuvant anti-tumor treatment. Consistent with our study, overexpression of 14-3-3ζ stratified a subgroup of patients with reduced survival in hepatocellular carcinoma, head and neck squamous cell carcinoma, and breast cancer [35, 36]. 14-3-3ζ was found to cooperate with other well-established oncogenes (such as ErbB2) to drive a more aggressive malignant phenotype in breast cancer [27]. This could be recaptured in NSCLC because the mutational burden of NSCLC is much higher and the mutational landscape is far more complicated. For instance, biological experiments have demonstrated that EGF treatment leads to the cell membrane recruitment of 14-3-3ζ and binding to the C-terminal tail of EGFR [37]. This 14-3-3ζ and tyrosine kinase signaling interaction may have translational significance, in which co-targeting 14-3-3ζ and tyrosine kinase would completely block the cell survival advantage and enhance the clinical efficacy of anti-tumor treatment.

Our study also provided preliminary evidence highlighting how 14-3-3ζ promotes disease recurrence and tumor metastasis. We found that cancer cells overexpressing 14-3-3ζ exhibit increased motility and invasiveness. Because accumulating evidence shows that the detachment of cancer cells from the original tumor mass primes tumor invasion, dissemination and outgrowth, we anticipated that 14-3-3ζ and NSCLC metastasis converge at EMT. Interestingly, we readily detected the turnover of EMT markers after disrupting the expression of 14-3-3ζ. Briefly, NSCLC cells with high expression of 14-3-3ζ gain increased migration and invasion capacity by losing cell-cell adhesion, while siRNAs-mediated knockdown of endogenous 14-3-3ζ partially restores E-cadherin expression and prevents cell dissemination. One potential explanation for reduced E-cadherin expression in 14-3-3ζ overexpressing cells would be increased transcription of the E-cadherin repressors, Snail and Slug. These repressors bind to E-cadherin promoter and recruit corepressors, including histone deacetylase 1 (HDAC1) and HDAC2, to silence the expression of E-cadherin [38]. In agreement with this, scientists have discovered a high-affinity motif in the C-terminal tail of Snail protein binds to 14-3-3 proteins, and mutation within this region abolishes 14-3-3 binding and inhibits Snail-mediated epigenetic repression of E-cadherin [39].

The molecular mechanism underlying EMT in disease recurrence has not been fully understood. Previous studies have suggested EMT is implicated in the “cancer stem cell” traits that may initiate recurrent tumors from disseminated cancer cells [40]. In our study, we reported that cancer cells overexpressing 14-3-3ζ have an increased survival advantage by increasing the phosphorylation level of Akt. By inhibiting the expression of 14-3-3ζ, cancer cell proliferation and survival were markedly suppressed, probably through aberrant phosphorylation of Akt [18]. These data also imply a therapeutic significance for co-targeting 14-3-3ζ. As we have illustrated in this study, inhibiting 14-3-3ζ prevents cancer cell migration and invasion, and sensitizes chemotherapy, thus, 14-3-3ζ would be recognized as both prognostic and therapeutic targets for patients with NSCLC. Co-targeting 14-3-3ζ or its downstream executors, including phosphorylated Akt, serves as novel treatment options for NSCLC.

Taken together, our data suggest that overexpression of 14-3-3ζ has an oncogenic role in NSCLC. Patients with high expression of 14-3-3ζ are at a high risk of disease recurrence and may be resistant to chemotherapy. Targeting 14-3-3ζ inhibits cancer cell migration and invasion by disrupting EMT program, and sensitizes cancer cells to chemotherapy-induced apoptosis. The development of novel 14-3-3ζ inhibitors is expected to be an effective strategy for patients whose tumor overexpresses 14-3-3ζ and are at a high risk of disease recurrence and metastasis.

## MATERIALS AND METHODS

### Patients and samples

Formalin fixed paraffin-embedded (FFPE) surgical resected samples were obtained from 22 patients who were diagnosed with stage II and stage IIIA NSCLC from 2019 to 2020 in our hospital. These patients received adjuvant chemotherapy (pemetrexed and platinum) after pulmonary lobectomy with lymph node dissection. Paired tumor samples and adjacent non-tumor samples were collected and processed in compliance with protocols approved by the ethic committee of Jinling Hospital, Nanjing University School of Medicine.

### Immunohistochemistry

To determine the expression of 14-3-3ζ, E-cadherin and vimentin in the NSCLC samples, immunohistochemistry (IHC) was performed following standard protocol. Briefly, 5 μm thick FFPE sections were prepared in xylene and graded ethanol. Antigen retrieval was done by boiling the slides in antigen retrieval buffer. Endogenous peroxidase was quenched in 0.3% H_2_O_2_. The slides were incubated with corresponding primary antibodies overnight in 4°C refrigerator. The slides were then incubated with HRP-conjugated secondary antibodies at room temperature for 1 h, visualized using a DakoEnVision Detection Kit (Dako), and counterstained with hematoxylin. IHC staining of 14-3-3ζ was set as negative, weak positive, moderate positive, and strong positive based on percentage of cells staining positive and staining intensity. Staining was scored by two independent pathologists and all cases were scored without knowledge of the clinical-pathological or outcome data.

### Cell culture

Human NSCLC A549, H358, H292, H1299 and SPC-A-1 cells were purchased from the cell bank of Shanghai Institute of Biochemistry and Cell Biology. The HCC827 and H3122 cells were generously gifted by Jeffrey Engelman (Massachusetts General Hospital Cancer Center/Biomedical Institute of Novartis). The Lewis lung cancer (LLC) cells were generously gifted by Junlong Zhao from Fourth Military Medical University. Cells were tested for mycoplasma contamination within the last 6 months and were routinely cultured in RPMI-1640 medium supplemented with 10% fetal bovine serum (Gibco), 100 U/ml penicillin and 100 μg/ml streptomycin (Corning) in a humidified atmosphere with 5% CO_2_ at 37°C.

### Plasmids and siRNAs

HA-tagged full-length 14-3-3ζ cDNA was PCR amplified and constructed into the CMV promoter-derived mammalian expression vector (LV011-puro, Hanbio Biotech). The lentivirus packing plasmids psPAX2 and pMD2.G have been extensively described and preserved in our in-house construct bank. All plasmids have been thoroughly sequenced.

siRNA targeting 14-3-3ζ were synthesized by RiboBio Biotech, the 14-3-3ζ siRNA sequence was listed as follows: Sense: 5′-CGCUGGUGAUGACAAGAAAdTdT-3′, Anti-sense: 5′-UUUCUUGUCAUCACCAGCGdTdT-3′.

### siRNAs transfection, lentivirus packing and generation of stable cell lines

Cells were seeded at the density of 2.5 × 10^6^ cells/ml in a 6 cm dish. After attachment, cells were transfected with indicated siRNAs using Lipofectamine 2000 (Invitrogen) according to the manufacturer’s recommendations. Forty-eight hours after transfection, cells were used for further experiments.

14-3-3ζ lentivirus was produced by transfecting HEK293 cells with LV011-14-3-3ζ construct together with the packaging plasmids psPAX2 and pMD2.G using Lipofectamine 2000. Culture supernatants containing lentivirus particles were collected at 48 and 72 h after transfection and stored at −80°C. Cells were infected with the lentivirus supernatants and selected with 2 μg/mL puromycin (Sigma).

### Cell proliferation assay

Cells were seeded at a density of 5 × 10^3^ cells/well in 96-well culture plates. Cells were incubated in the culture chamber overnight to allow attachment. After that, cells were treated as indicated. Sterile MTT solution (Sigma) was added, and the cells were incubated for an additional 4 h. The supernatant in each well was carefully removed and formazan crystals were dissolved in 150 μl DMSO for 10 min with gentle shaking. The absorbance at 570 nm was recorded using an enzyme-linked assay reader (Bio-Rad).

To determine the significance of 14-3-3ζ on cancer cell proliferation at a relatively longer duration, colony formation assay was performed as we have previously described. Cells were seeded into 6-well plates at a density of 1 × 10^3^ cells per well and allowed to grow freely in the medium for approximately 14 days. The medium was refreshed every 3 days. At the end of the experiment, cell colonies were fixed with 4% paraformaldehyde and visualized by crystal violet staining.

### Scratch healing assay

Cells were cultured into 6-well plates. An artificial wound was created by manually scraping the monolayer when cells grew over 80% confluence. Photographs were taken at 0, 24, 48 h, respectively, and five fields were taken randomly.

### Migration and invasion assays

The transwell (Corning) experiment was performed to assess the migration capacity of cells after manipulating 14-3-3ζ. Briefly, cells were serum starved for 12 h and resuspended at a final concentration of 2 × 10^4^ cells/ml with serum-free RPMI-1640 media (Corning). Cells were seeded into the upper chamber and RPMI-1640 media with 10% FBS was added in the lower chamber. After incubation for 24 h, migrated cells which penetrated the membrane were fixed with 10% methanol for 30 min and stained with Giemsa (Sigma), whereas cells on the upper surface of the membrane were carefully removed by cotton swabs. Migrated cells were counted under a light microscope at 100× magnification. For the cell invasion assay, the polycarbonate membrane was coated with 100 μl Matrigel (BD BioCoat Matrigel). The following assay was similar to that described in the transwell migration assay. All experiments were repeated three times.

### qPCR assay

Total RNA was extracted with the TRIzol reagent (Invitrogen). First-strand cDNA was prepared from 1 μg of total RNA and qPCR was performed following instruction (Takara). The relative amount of each gene was measured by real-time qPCR experiment and calculated using the 2^−ΔΔCT^ method. The qPCR primers sequences were listed as follows:

E-cadherin Forward: CGAGAGCTACACGTTCACGG; E-cadherin Reverse: GGGTGTCGAGGGAAAAATAGG; Vimentin Forward: GACGCCATCAACACCGAGTT; Vimentin Reverse: CTTTGTCGTTGGTTAGCTGGT; Snail Forward: ACTGCAACAAGGAATACCTCAG; Snail Reverse: GCACTGGTACTTCTTGACATCTG; Slug Forward: TGTTGCAGTGAGGGCAAGAA; Slug Reverse: GACCCTGGTTGCTTCAAGGA; β-actin Forward: CATGTACGTTGCTATCCAGGC; β-actin Reverse: CTCCTTAATGTCACGCACGAT;

### Western blot analysis

After the indicated treatment, cells were harvested and lysed in lysis buffer. Equal amount of proteins (10–30 μg) were separated by SDS-PAGE and transferred onto a nitrocellulose membrane (Millipore). The membranes were then blocked with 5% non-fatty dry milk in TBST for 2 h and probed overnight at 4°C with primary antibodies against the following proteins: 14-3-3ζ (1/1000, Abcam), E-cadherin (1/1000, Cell Signaling Technology), vimentin (1/1000, Cell Signaling Technology), Snail (1/1000, Cell Signaling Technology), Slug (1/1000, Cell Signaling Technology), Bcl-2 (1/1000, Cell Signaling Technology), Bcl-xL (1/1000, Cell Signaling Technology), MCL-1 (1/1000, Cell Signaling Technology), Bax (1/1000, Cell Signaling Technology), PARP (1/1000, Cell Signaling Technology), cleaved caspase-3 (1/1000, Cell Signaling Technology) and β-actin (1/2000, Cell Signaling Technology). The membranes were washed in TBST and then incubated in HRP-conjugated secondary antibodies (1/5000, Cell Signaling Technology) for additional 1 h. The specific protein bands on the membranes were visualized by enhanced chemiluminescence (Millipore).

### Animal housing and care

The adult male C57BL/6J mice were purchased from Qinglongshan Animal Breeding Field (Nanjing, China). Mice were housed in pathogen-free condition with sterile woodchip bedding supplied with sterile food and water. Mice were kept in a room maintained at temperature of 21–24°C on a scheduled 12 h light/dark cycle.

### LLC cells tail vein injection

A total of 16 mice, 20–30 g in weight, were divided into two groups and received LLC NC siRNA and LLC 14-3-3ζ siRNA injection, respectively. Briefly, mice were restrained and injected into the medial tail vein with 1 × 10^5^ indicated cells suspended in 100 μl of phosphate buffer saline (PBS). Mice were weighed and monitored for mobility, respiratory distress, and signs of pain daily for up to 8 weeks. A weight loss of more than 20% was deemed to be unacceptable and would lead to early sacrifice of the mouse. At the end of experiment, the mice were then humanely sacrificed, and lungs were carefully isolated and processed for histologic studies. All animal experiments were conducted in compliance with institutional guidelines and approved by the Institutional Ethics Committee of Jinling Hospital.

### Statistical analysis

All experiments were performed at least in triplicate and the results are presented as the mean ± standard deviation. Data were analyzed by using the SPSS software (version 10.0) and GraphPad Prism 5.0 software. Statistically significant differences among groups were determined using one-way ANOVA and Dunnet’s least significant difference post hoc tests. *P* < 0.05 was considered to indicate a statistically significant difference (^*^P < 0.05, ^**^P < 0.01, ^***^P < 0.001).
